# Supplementary material for: Right hemisphere atrophy rate associates with naming recovery after left hemisphere ischaemic stroke
Source: Brain Commun. 2026 Jul 27;8(4):fcag291. doi: 10.1093/braincomms/fcag291 (PMC13439314; doi:10.1093/braincomms/fcag291)
Supplement: Supplementary file 1 [file fcag291_supplementary_data.zip › Supplementary_material.pdf]

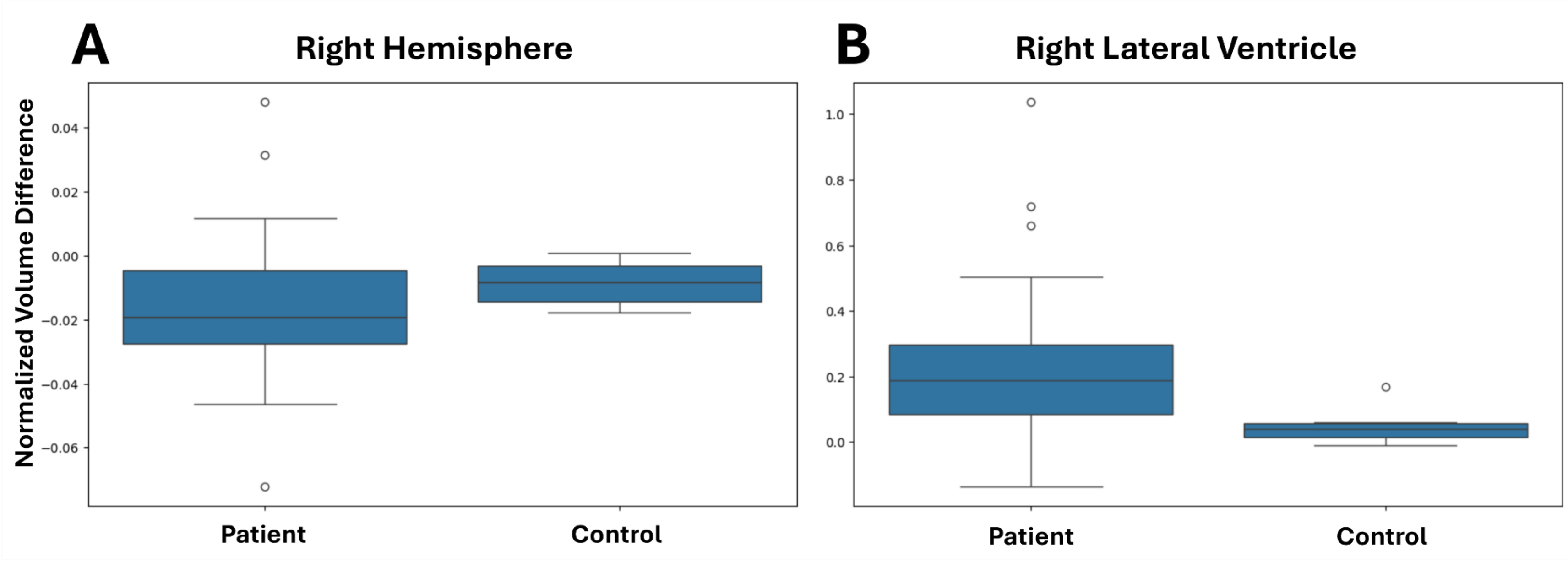

Supplementary Figure 1 Longitudinal changes in brain volumes. Groupwise comparisons were conducted using two-tailed unpaired t-tests. Each dot represents a single patient. There was a tendency for right hemispheric volume to decline (**A**) and ventricular volume to increase (**B**) in both controls (n=11) and patients (n=72), but there was greater right hemispheric volume decrease (p=0.028) and greater right lateral ventricle volume increase (p=0.006) in patients compared to controls.

Supplementary Table 1 Demographics and Naming Scores for Participants Removed from Figure 3B

	Stroke Survivors (*n* = 28)	
	***M* (*SD*)**	**Range**
Age (years)	60 (11)	37 to 79
Education (years)	15 (3)	9 to 20
Lesion Volume (cc)	4.97 (7.98)	0.31 to 37.38
Naming Performance		
Acute BNT Score (/30)	27 (2)	25 to 30
Chronic BNT Score (/30)	28 (2)	22 to 30
Change in BNT Score	1 (2)	-3 to 3
Sex (Female:Male)	15:13	
Race (Black:White)	16:12	
Ethnicity (Non-Hispanic:Hispanic)	27:1	
Handedness (Left:Right)	3:25	

BNT = *Boston Naming Test – Shortened*.[1,2]

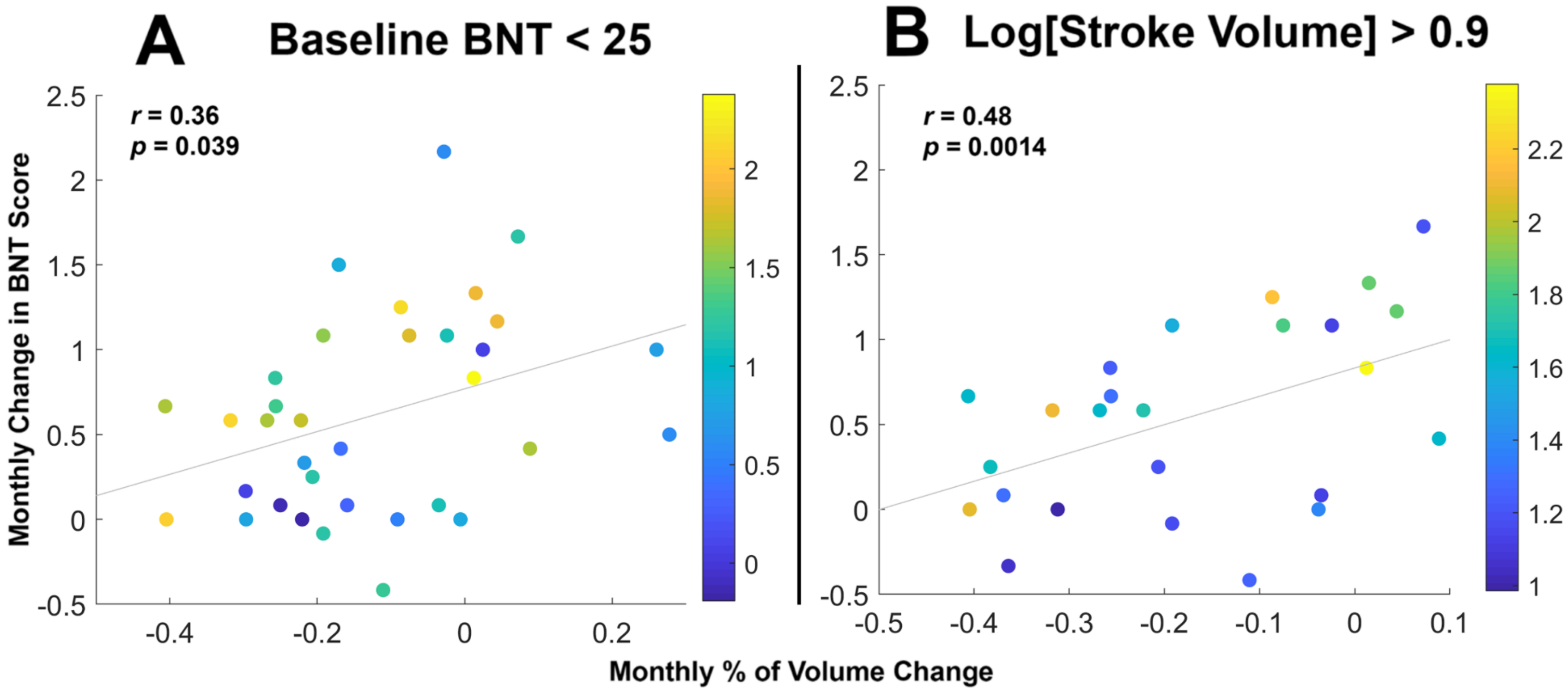

Supplementary Figure 2 Scatter plots of right hemisphere atrophy versus change in naming. The relationship between right hemispheric atrophy as a monthly percentage and monthly change in naming score on the Boston Naming Test was assessed using Pearson's correlation. Each dot represents a single patient, with lines representing the fitted regression. Patients with greater atrophy tend to show less recovery, a trend that is maintained when excluding both high performers at baseline (**A;** r=0.36; p=0.039; n=34) and those with smaller strokes (**B;** r=0.48; p=0.0014; n=25). The color scales shows Log[Stroke Volume] in mL.

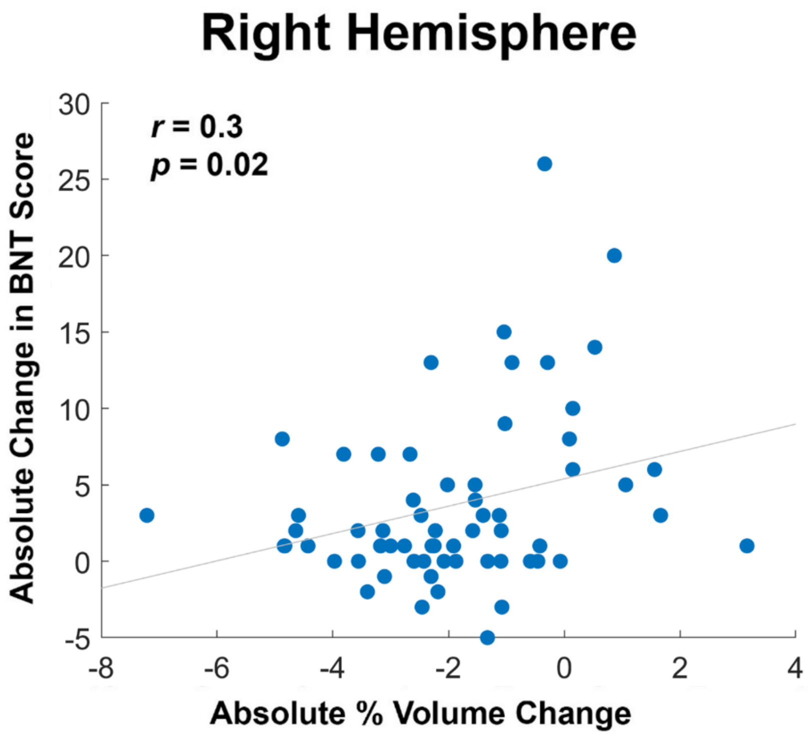

Supplementary Figure 3 Scatter plot of absolute right hemisphere atrophy versus absolute change in naming. The relationship between right hemispheric atrophy as an absolute percentage and absolute change in naming score on the Boston Naming Test was assessed using Pearson's correlation; n=62. Each dot represents a single patient, with a line representing the fitted regression. Atrophy of the right hemisphere was associated with worse recovery of naming (r=0.3; p=0.02).

References

1. Kaplan E, Goodglass H, Weintraub S. *Boston Naming Test*. Pro-Ed; 2001.
2. Mack WJ, Freed DM, Williams BW, and Henderson VW. *Boston Naming Test*: Shortened Versions for Use in Alzheimer's Disease. *J Gerontol.* 1992;47:154–158.
